# Removal of benzene, MTBE and toluene from contaminated waters using biochar-based liquid activated carbon

**DOI:** 10.1038/s41598-022-24283-6

**Published:** 2022-11-16

**Authors:** F. Alshahrani, B. Tawabini, T. Saleh, M. Alrayaan, S. Alaama, R. Nasser, P. Soupios, P. Kirmizakis, M. Mahmoud, T. Oyehan, E. Safi

**Affiliations:** 1 Department of Geosciences, College of Petroleum Engineering & Geosciences, King Fahd University of Petroleum & Minerals, Dhahran, 31261 Saudi Arabia; 2 Department of Chemistry, King Fahd University of Petroleum & Minerals, Dhahran, 31263 Saudi Arabia; 3 Saudi Arabian Oil Company, Dhahran, Saudi Arabia; 4 Department of Petroleum Engineering, College of Petroleum Engineering & Geosciences, King Fahd University of Petroleum & Minerals, Dhahran, 31261 Saudi Arabia

**Keywords:** Environmental chemistry, Chemical engineering, Civil engineering

## Abstract

Fuel components such as benzene, toluene, and methyl tertiary-butyl ether (MTBE) are frequently detected pollutants in groundwater resources. Ex-situ remediation technologies by activated carbon have been used for treatment for many years. However, due to high cost of these technology, more attention has been given to the in-situ remediation methods of contaminated groundwaters using liquid carbon adsorbents. Literature search showed limited studies on using adsorbents in liquid form for the removal of such contaminants. Therefore, this lab-scale study investigates the capacity of using raw biochar-based liquid activated carbon and iron-modified biochar-based liquid activated carbon to remove these pollutants. The adsorption efficiency of the synthesized liquid activated carbon and iron-modified liquid activated carbon mixed with sand, limestone, and 1:1 mixture of sand/limestone, was tested using batch suspension experiments. Adsorption by granular activated carbon was also investigated for comparison with liquid activated carbon. Results of the study revealed that mixing of liquid activated carbon or LAC-Fe on subsurface materials had not improved the removal efficiency of MTBE. At the same time, it showed a slight improvement in the adsorption efficiency of benzene and toluene. In all cases, the removal by GAC was higher with around 80% and 90% for MTBE and BT, respectively. Results also showed that benzene and toluene were better removed by liquid activated carbon and iron-modified liquid activated carbon (∼ 40%) than MTBE (∼ 20%). It is also found that water chemistry (i.e., salinity and pH) had insignificant effects on the removal efficiency of pollutants under the study conditions. It can be concluded that more research is needed to improve the capacity of biochar-based liquid-activated carbon in removing MTBE, benzene and toluene compounds that will lead to improve the utilization of liquid activated carbon for the in-situ remediation of contaminated groundwaters.

## Introduction

Fuel compounds such as benzene, toluene, ethylbenzene, and xylene (a.k.a BTEX) and fuel additives such as methyl tertiary-butyl ether (a.k.a MTBE) are well-known pollutants that may cause serious environmental and health issues if not removed from the water before use. BTEX and MTBE are highly mobile and soluble in groundwater, regulated up to 5 μg/L and 13 μg/L in drinking water^1,2^. MTBE is substantially more soluble than BTEX, sorbs less, is less likely to volatilize out of groundwater, and is less likely to be biodegraded^3,4^. For the last decades, BTEX removal has become a focus as it is toxic and carcinogenic even at low concentrations^5^. In the 2000s, the United States and Canada banned MTBE use^6^ after a study reported its widespread occurrence in shallow groundwater^7^. However, the recent call for reducing carbon emissions appears to favor the increased use of MTBE, and its production is predicted to continue growing through 2050^8^. Wide treatment applications have been applied for MTBE & BTEX remediation, including physical, chemical and biological methods^9–15^. MTBE adsorption by activated carbon (AC) was studied, and it found high solubility of MTBE in water is a disadvantage of granular activated carbon (GAC) adsorption and took around 15 h for total removal^16^. Thermally modified diatomite was investigated^17^ to adsorb MTBE & BTEX and found the highest adsorption by modified diatomite at 550 °C and lower adsorption at 750 °C and 950 °C. For effective sorption of MTBE on activated carbon, the AC surface should be tailored to have sufficient small diameter mesopores having pore volumes within the range of 14–200 Å^18,19^. Achieving these pore size limits suggests reducing the particle size to minute size ranges. Pan et al.^20^ reported that AC particle size range of as low as 140 nm is favourable for removing hydrophilic and high molecular weight organic compounds. Nanomaterials have smaller size ranges and have so far proved effective in the sorption of many contaminants in pump-and-treat and (field) aquifer remediation^21,22^. MTBE adsorption via hydrophobic zeolites (silicalite, dealuminated Y, mordenite, and beta) wasinvestigated^23^ and found to perform better than GAC, particularly in the μg/L range. In another study, activated carbon was obtained from five residues for BTEX adsorption and found low adsorption maybe due to porosity or surface chemical parameters they contained acid^24^. Thermally treated lignite at several temperature ranges was studied into batch adsorption experiment and found effective adsorption of MTBE, BTEX & TAME (tertiary amyl methyl ether) at lignite 750 °C^25^. Another MTBE removal study by composites of polyacrylamide (PAM)-zeolite than untreated zeolite and GAC found efficient MTBE treatment^26^. BTEX adsorption tested by rice husk and found effective^27^.

Moreover, ordered mesoporous carbon (OMC) proves the high efficiency of BTEX adsorption^28^. Organo-sepiolite material was found to be a high-potential adsorbent for BTEX adsorption^29^. Another study for MTBE treatment by GAC adsorption found practical and high operation costs compared with air stripping and advanced oxidation^30^. Peat and angico hardwood sawdust^31^ showed good potential for BTEX adsorption from produced water. Ordered Mesoporous Carbon (OMC)^32^ was found to have 27% higher efficiency than GAC. BTEX adsorption by montmorillonite modified^33^ with polyethylene glycol was investigated, and found good adsorption at 200% cation exchange capacity within 24 h. Smectite organoclay for a single-solute system to adsorb Benzene was investigated and found adsorption in the range of 55–90% adsorption^34^. BTEX adsorption was investigated via biochar from palm pits impregnated with ferrous (III) chloride (FeCl_3_)^35^ and found high adsorption efficiency with increasing pH, contact time, and adsorbent amount.

Biochar is the solid, porous, carbonaceous material produced by biomass pyrolysis in the temperature range of 300–800 °C^36–38^. The combination of biochar production conditions, pyrolysis method, and feedstock material result in distinctively different physical and chemical properties^39^. These properties control the behavior and performance of biochar in various applications, such as an additive in soils, soil remediation, and wastewater treatment^40,41^. In wastewater treatment, biochar has been mainly used either as a reusable adsorbent of contaminants or as a substrate for developing catalysts for the oxidative degradation of organic substances^42^. Another interesting study by Date Palm was found a promising adsorbent for many pollutants in aqueous solution^43^ and it needs further research for an adsorption–desorption-re-adsorption approach to achieve a zero waste strategy. The impact of surface modification on the adsorptive removal of BTEX onto solid carbonaceous material was studied and achieved sorption equilibrium within 30 min^44^. Adsorption technology on alarge scale is expensive; alternative low-cost adsorbents draw researchers’ attention. Their manufacturing takes around 1–2 days with a 15–120 min adsorption process duration^45^. Another investigation by sulfonated carbon (SC) using the H_2_SO_4_ was found in higher adsorption than mesoporous Carbon synthesized under different hydrothermal ranges^46^. Moringa Oleifera Seeds and Banana Peel^47^ were investigated and compared with GAC and found promising adsorbents.

Similar to nanoparticles, -activated carbons (LAC) are currently being used and investigated as adsorbents for in situ groundwater treatment. For instance, in 2015, Georgi et al.^48^ prepared LAC and injected them into the subsurface to study its mobility within the aquifer. While the mobility depended on the chemical stabilizers used for making the LAC, the LAC’s efficacy in remediating organic contaminants was only demonstrated in a column batch study—the deposited LAC effect treatment from within the sediment. Within the same year of Georgi’sresearch, a patent on liquid-activated carbon (PlumeStop^®^) was filed^49^. The patented liquid AC has a dual function; working like colloids by adsorbing contaminants as it passes through soil and groundwater after injection and providing a high surface area matrix favorable for microbial colonization and growth. Intrinsic biodegradation can be further enhanced with the proximal co-application of extended oxygen release for enhanced aerobic biodegradation. Promising results have also been presented by Mackenzie et al.^50^ developing LAC impregnated with zero-valent iron(ZVI) (CARBO-IRON^®^) for dechlorination treatment in an aqueous solution.

Nonetheless, in-situ groundwater remediation is more complicated than just injecting an adsorbent. All contaminated aquifers are not the same. The differences in geology, transport distribution, contaminant types and mass, and biological activities in the polluted sites could impact any adsorbent’s efficiency and effectiveness during groundwater remediation^51^. For these reasons, some recent studies were conducted to investigate PlumeStop products' efficacy to remediate per- and polyfluoroalkyl substances (PFAS) in specific aquifer types in Canada^52,53^ and Sweden^54^. So far, this novel liquid AC product’s efficiency for in-situ remediation of organic contaminants in different locations and different contaminants is still poorly documented. No report was found for the application of liquid or colloidal AC in the Middle East. Also, neither the manufacturer nor any other researcher has ever reported the liquid adsorbent’s performance when there are multiple contaminants, such as BTEX and MTBE. Therefore, the objectives of this study are; to examine the efficiency of locally synthesized LAC and iron-modified LAC (LAC-Fe) on the remediation of both MTBE and BTEX in groundwater; to optimize at laboratory scale, the treatment parameters such as pH, LAC dose, conductivity, contaminants concentration; and to investigate the role(s) of the tested parameters on the remediation performance of the LAC in MTBE and BTEX treatment.

## Materials and methods

### Synthesis of biochar-based liquid activated carbon (LAC)

Palm fibers were locally collected, cleaned, grinded, and then separated by a sieve to a fine–size of 1–2 mm in diameter. The sieved fibers were then carbonized at 450 °C for 300 min under a flow of nitrogen (99.9%) flow in a stainless steel vertical tubular reactor placed in a tube furnace. 5% hydrogen peroxide (H_2_O_2_) solution with a ratio 1:10 wt%, char: H_2_O_2_ was added to the prepared char. The mixture was flushed with nitrogen gas (99.9%) to remove the air and oxygen. After 10 h of stirring, the mixture was filtered. Then, the produced carbon black was further modified with oxygen-containing groups by the treatment with nitric acid (1.0 M HNO_3_) in a ratio of 20 mL (HNO_3_) to 1.0 g Carbon. The mixture was then heated up to 90 °C, kept under reflux, and stirred for 3 h after which it was allowed to cool down to room temperature. After that, it was filtered and washed with distilled water several times. The synthesized biochar-based activated carbon was then liquefied by mixing with distilled water at a ratio 1:10 wt%, then it was sonicated for 1 h. After that, 0.5% polyethylenimine was added into the mixture, and the system was further sonicated for 5 h to obtain liquid activated carbon (LAC). Table 1 below shows the main characteristics of the biochar.

**Table 1:** Biochar characteristics.

Production temperature (°C)	450
Residence time (min)	300
Total C (%)	86
N (%)	2
O (%)	12
O/C ratio	0.14
Ash content (%)	5
pH	6.5
Conductivity (μS cm^−1^)	38
Surface area (m^2^ g^−1^)	786
Total pore volume cm^3^/g	0.68

For LAC modification with iron (Fe), around 10 g of carbon was dispersed in a solution of 150 ml of distilled water, and 100 ml of ethanol, in the presence of 10 ml of diethylene glycol. After that, the components were kept under stirring for 8 h. Then, 3.4 g of hydrous ferrous (II) sulphate heptahydrate (FeSO_4_·7H_2_O) dissolved in 20 ml water was drop-wise introduced into the system. Then, 8.8 g of ferric (III) chloride hexahydrate (FeCl_3_·6H_2_O) dissolved in 20 ml water was added. The system was then kept under stirring for 4 h. After that, pH was adjusted to > 7 using 0.1 M ammonia solution. After that, the temperature was adjusted to 100 °C with stirring for 8 h. Then, the system was allowed to cool. The produced iron-modified carbon was collected.

### Chemicals and materials

Benzene (anhydrous, 99.8%), (MTBE (p.a., > 99.5% GC), toluene (ACS, 99.7%) compounds were purchased from Sigma Aldrich.ACS (American Chemical Society standards) reagent0.1 M hydrochloric acid and 0.1 M sodium hydroxide solutions were used for pH adjustment. The Millipore Aquinity P70 Water Purification System prepared distilled water in the lab. A 1000 ppm stock mixture of the three above compounds were prepared by dissolving 1.0 gm in 1L distilled water and stirring for 3 h until the compounds were completely dissolved in the solution. The spiked solutions of 2000 μg/L (or 2 ppm) mixture of MTBE, Toluene, and BTEX were prepared fresh for every batch of treatment runs by diluting the stock mixture in distilled water at a ratio of 1:500. Other spiked solutions were prepared in water of different salinity (i.e., 5000 and 10,000 μS/cm).

Three (3) types of host porous materials (sand, limestone, and 1:1 mix) were used in the study to represent the subsurface material that simulates the condition when LAC is injected in the situ-remediation methods. Test (Ottawa) sand H-3825 purchased from Humboldt Co., USA. A bulk-size limestone rock collected from a local site in Dhahran, Saudi Arabia, was crushed into power-size using a crusher. The limestone crushed sample was sieved to a size of 1.4 ≤ 2 mm. The mixture of OS and LS was prepared by mixing 5 g from each material to obtain a 50/50 mix. Commercial granular activated carbon (GAC) with a 40/60 mesh less than 2 mm size was purchased fromGharbalah Industrial Co, Riyadh, Saudi Arabia, and used in the study for comparison purposes.

### Apparatus and chemical analysis

The water samples were analyzed according to EPA Method 8260^55^ by Trace GC Ultra ISQ Gas Chromatography/Mass Spectrometry (GC/MS) manufactured by Thermo Scientific. The GC/MS was equipped with a TriPlus HS headspace analyzer. At each sampling interval, 1 mL of the solution was collected in 5 ml vials and transferred to the GC/MS for analysis. The GC/MS is equipped with a headspace injection unit where solutions were incubated in agitator for 5 min at 90 °C, then volatile components were directly injected into the GC column by a headspace syringe heated at 120 °C. Restek Rtx-502.2 capillary column 60 m × 0.32 mm × 1.8 µm was used for the separation of volatile components. The GC was programmed at an initial temperature 40 °C for 2 min, then raised from 165 °C at a rate of 5 °C/min, then raised to 250 °C at a rate of 30 °C/min, and holding time of 5 min. The total run time on GC is 35 min. Selected ion monitoring (SIM) mode was used for the identification and quantitations of the analytes.

### Experimental work

For each run, 100 mL capped conical beakers filled with pollutants-mix spiked solution. Ten (10) g of material (OS, LS, and 1:1 OS/LS) were mixed thoroughly with 3 g of LAC & LAC-Fe to prepare the adsorbents. The prepared materials were added to the spiked solution and shacked in a shaker for around 2 h at 150 rpm. Then, 1 mL samples were taken from each beaker at 0, 10, 15, 30, 45, and 60 min, for analysis by GC/MS. Initially, the treatment runs were carried out using distilled water at pH 7. The spiked samples were first stirred for about 1 h to ensure the solubility of the organic pollutants in the water. Blank samples (i.e., materials without LAC) were included in the test runs to account for any adsorption (loss) due to the earth materials only. To assess the effect of pH and salinity, aset of adsorption runs were done at different pH of 4 and 10 and different EC (conductivity) of 5000 and 10,000 μS/cm, respectively. The brackish waters were prepared by diluting seawater (46,200 μS/cm) in distilled water at specific ratios. On the other hand, waters of different initial pH levels were prepared by adjusting the pH to approximately 4, 7, and 10 with HCl and NaOH.

## Results and discussion

### Characterization of carbons

**Figure 1:**
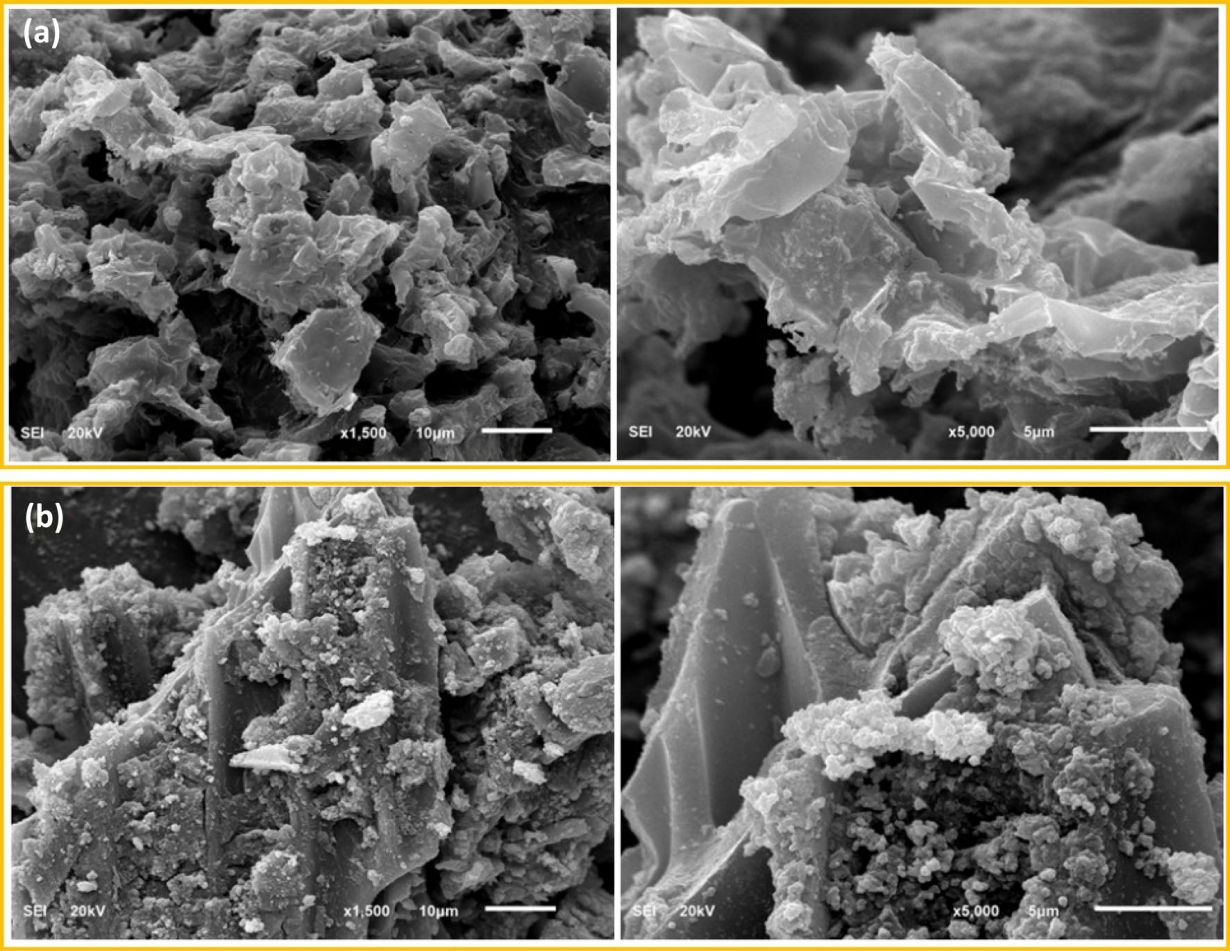
SEM images of synthesized carbons (top), and iron-modified carbon (bottom).

**Figure 2:**
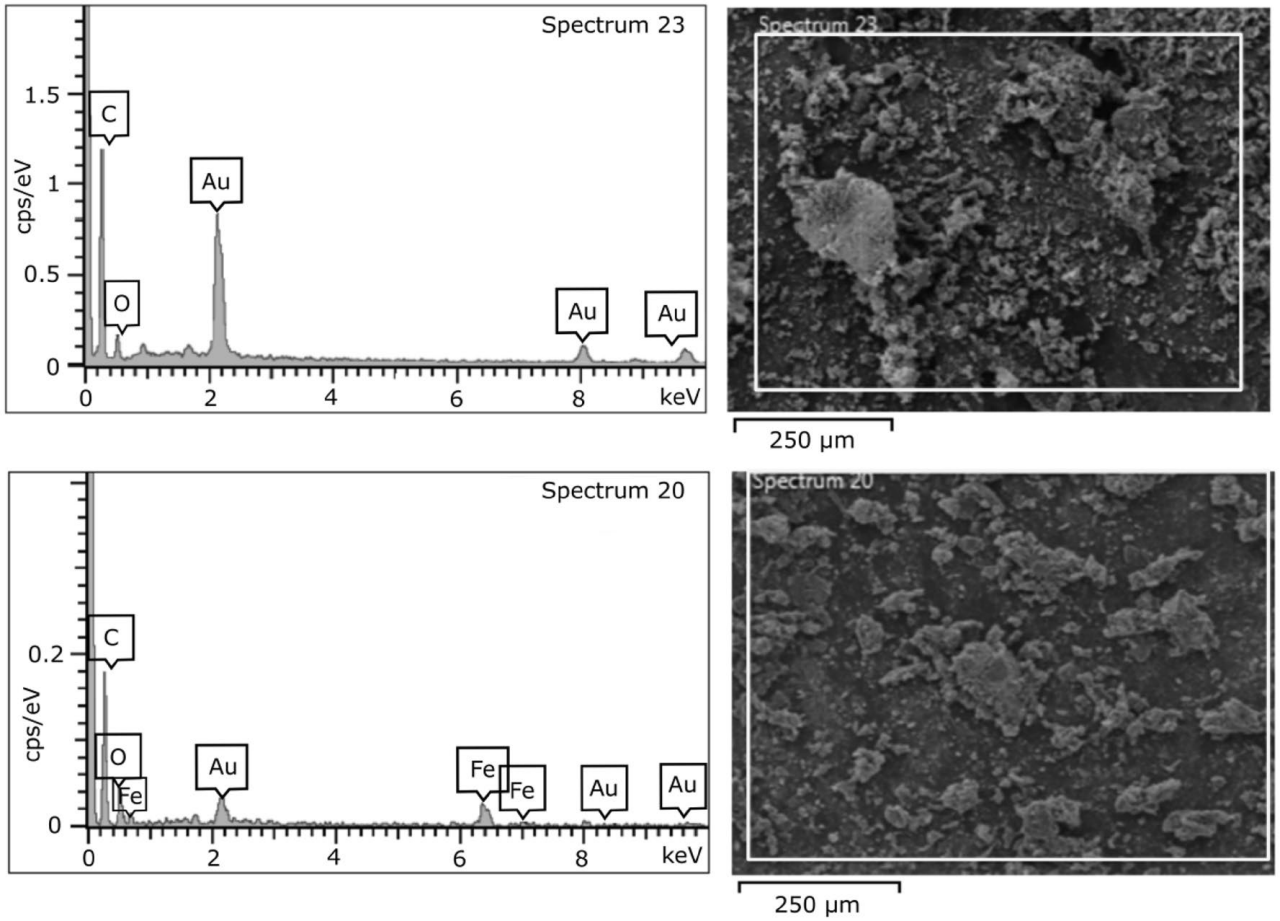
EDX spectra of synthesized carbons (top), and synthesized iron modified carbon (bottom).

Figure 1 displays the SEM images of the synthesized carbon and iron-modified carbon. SEM images of the carbon designate the slides-like shape of the prepared carbon. The surface is free of any dots or metal particles compared with the iron-modified carbon shown in the SEM images in Fig. 1. The increase in surface roughness is expected to change the surface properties after introducing iron oxide particles. Furthermore, the EDX spectra of the carbon and iron-modified carbon are presented in Fig. 2. As shown in Fig. 2, the main elements of the prepared carbon are carbon and oxygen, which indicates the presence of the oxygen functional groups on the carbon surface. At the same time, the EDX spectra of the iron-modified carbon are presented in Fig. 2. In addition to carbon and oxygen, iron is shown, indicating the formation of iron oxide on the surface of the prepared carbon.

**Figure 3:**
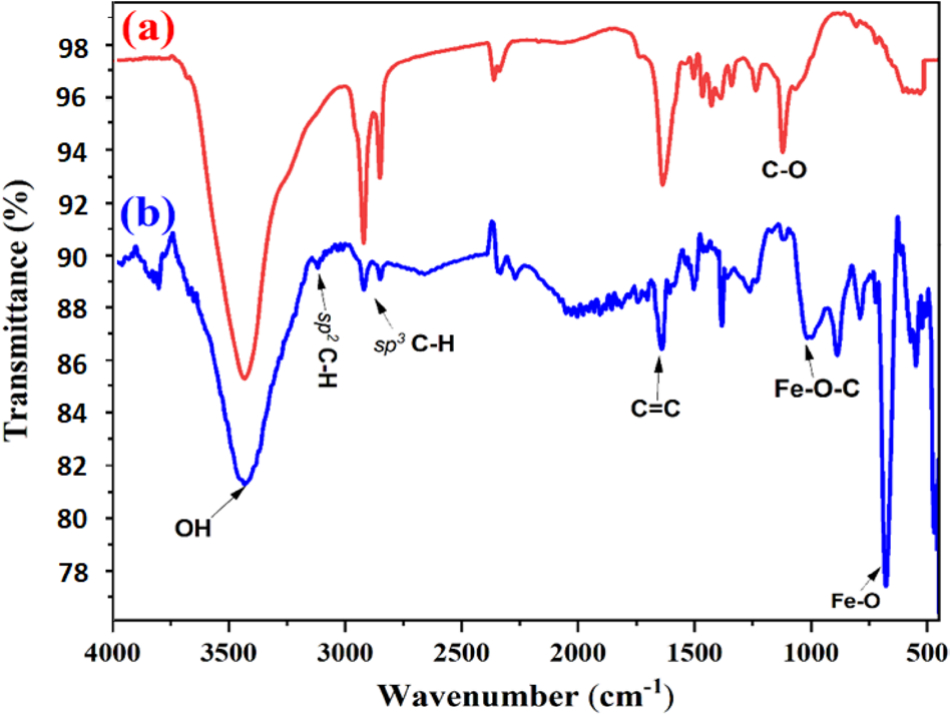
FTIR spectra of (**a**) liquid carbons and (**b**) iron-modified liquid carbon.

The FTIR spectra of the synthesized carbon and iron-modified carbon are shown in Fig. 3a,b, respectively. The band located at 1000 to 1100 cm^−1^ can be attributed to (–CO) stretching and (–OH) bending vibrations. The band at around 1450 cm^−1^ can be attributed to (CH_2_) bending. The bands at about 2920 and 2850 cm^−1^ are attributed to the bonds of C–H in CH and CH_2_ on the carbon structure. The band at about 2300 cm^−1^ is attributed to the C≡N bonds formed due to the treatment of carbon with nitric acid. The spectrum exhibits a band at ≈ 3400–3550 cm^−1^ attributed to OH stretching vibration. The bands between 1720 and 1600 cm^−1^ are assigned to carboxylic acids and carbonyl stretching vibration^56^. The bands appearing between 1450 and 1600 cm^−1^ are assigned to C=C aromatic from the carbon structure^57^. The band at about 1085 cm^−1^ is assigned to the Fe–O–C bonds^58^. The bands observed at 770 and 890 cm^−1^ are attributed to Fe–O bending vibrations. The band at 600 cm^−1^ is owing to the Fe–O stretching vibrations indicating the possible formation of Fe–O–C^59^.

### Removal of MTBE, benzene and toluene by uncoated materials (blank run)

**Figure 4:**
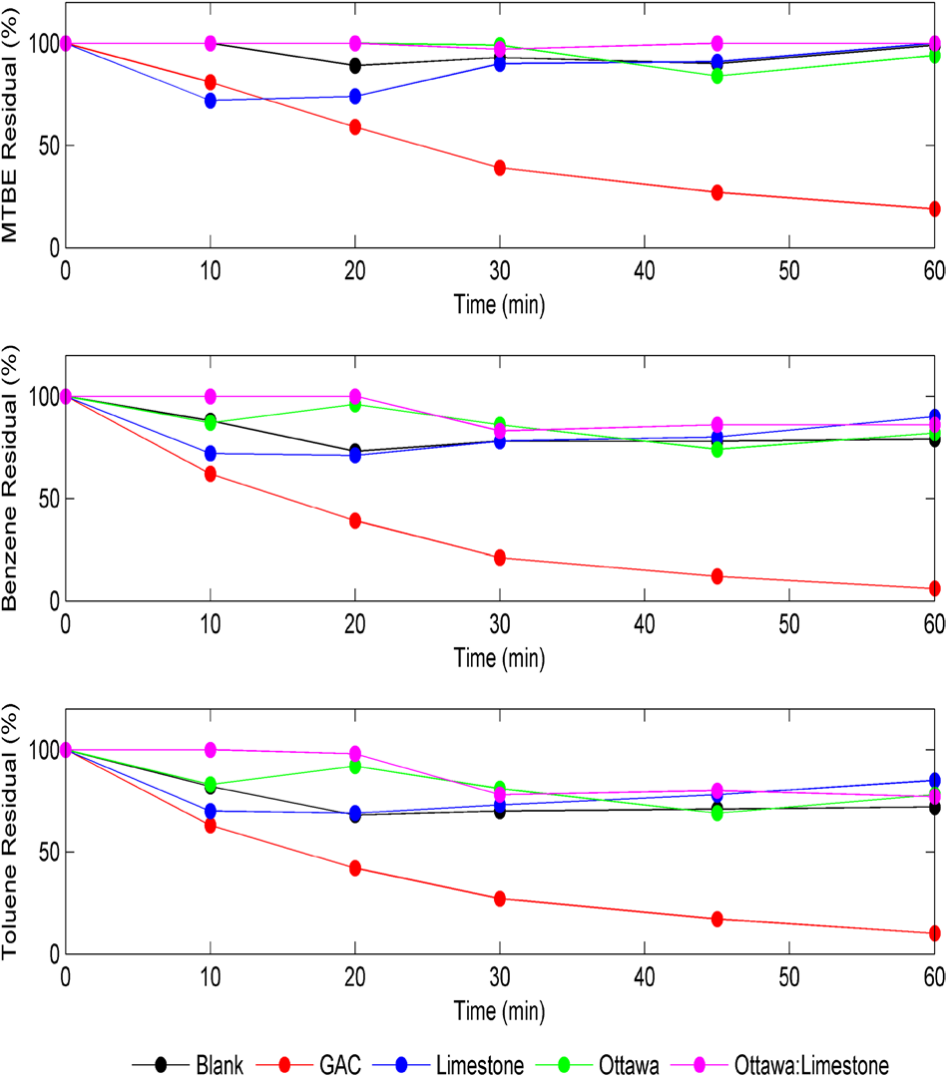
Removal of 2 ppm MTBE (top), benzene (middle) and toluene (bottom) using 10 gm uncoated materials and GAC at pH 7, and 150 rpm.

Figure 4 shows the removal of MTBE, Benzene, and Toluene, respectively, using natural uncoated materials (limestone (LS), sand (OS), and 1:1 LS/OS). In general, the results presented in Fig. 4 clearly show that the adsorption of the 3 pollutants by the uncoated materials was very low (< 20%) compared to adsorption by GAC which reached 80% for MTBE, 95% for Benzene and 90% for Toluene. The MTBE adsorption by LS shown in Fig. 4 showed slightly better removal than other materials in the first 20 min, after which the removal dropped again. This could be attributed to MTBE's solubility, which may vary during the stirring. Benzene removal was slightly better than MTBE, with around 20% removal achieved after 60 min for all three types of materials used. Toluene removal efficiencies showed similar removal efficiencies of Benzene.

### Removal of benzene, MTBE and toluene by different host materials coated with LAC

**Figure 5:**
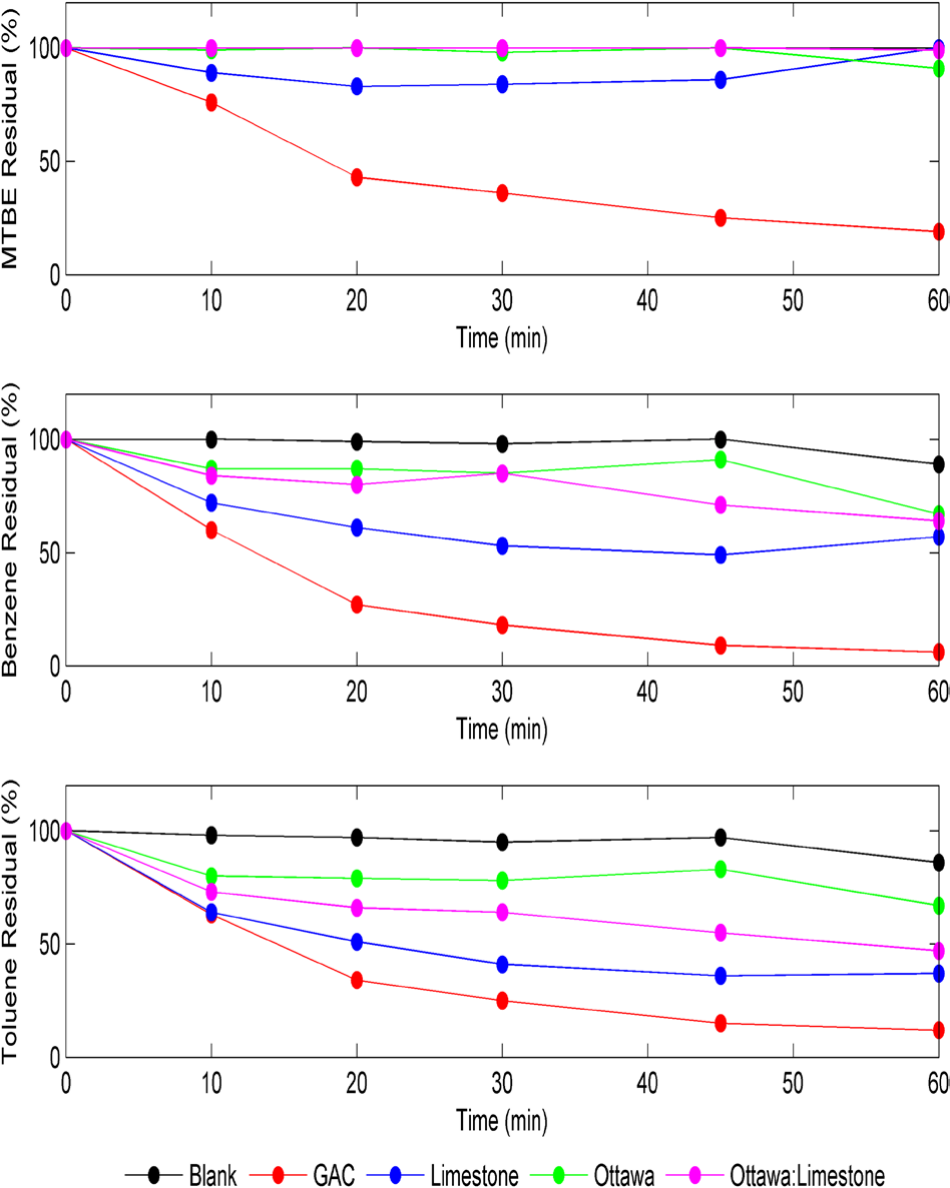
Removal of 2 ppm MTBE (top),benzene (middle) and toluene (bottom) using 10 g materials coated with 3 g LAC and GAC at pH 7, and 150 rpm.

The results of MTBE, benzene, and toluene removal efficiencies using unmodified LAC-coated materials (limestone (LS), sand (OS), and 1:1 LS/OS) are shown in Fig. 5. The results presented in Fig. 5 clearly show that adsorption of the three pollutants by the LAC-coated materials were generally low, compared to GAC, but slightly better (< 30%) than the uncoated materials. The main observation from Fig. 5 showed that LAC-coated limestoneenhancedthe adsorption for MTBE and Benzene. This maybe explains why more LAC has been adsorbed on the surface of the limestone compared to the solid impervious surface of the sand. On the other hand, the toluene removal pattern was different than both MTBE and benzene. In fact, the results shown in Fig. 5 revealed an improved removal efficiency of Toluene when the 3 materials were coated with LAC compared to the uncoated materials. For example, limestone coated with unmodified LAC increased the removal efficiency of Toluene to around 62% within 60 min, compared to only 18% when uncoated limestone materials were used, as shown in Fig. 4.

### Removal of benzene, MTBE and toluene by earth materials coated with LAC-Fe

**Figure 6:**
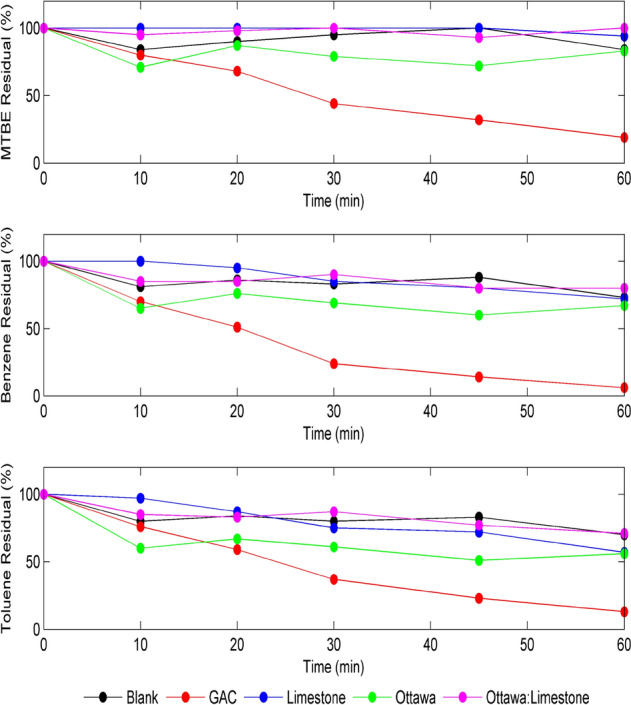
Removal of 2 ppm MTBE (top), benzene (middle), and toluene (bottom) using 10 gm materials coated with 3 g LAC-Fe and GAC at pH 7, and 150 rpm.

Figure 6 shows the removal of MTBE, Benzene, and Toluene, respectively, using LAC-Fe coated materials, limestone (LS), sand (OS), and 1:1 LS/OS. The results presented in Fig. 6 clearly show that the adsorption of the three pollutants by the LAC-Fe coated materials was generally low (30–40%) compared to adsorption by GAC which reached 80–95%. On contrary to what has been observed in Fig. 5, the MTBE adsorption by sand (Fig. 6) showed slightly better removal than other materials. The results indicated that the removal of MTBE slightly increased using either limestone coated with unmodified LAC or sand coated with LAC-Fe. This behavior could be explained by the fact that sand has a solid impervious surface compared to the permeable surface of limestone. The surface charges also play a role in this regard. Similarly, comparing the results of Figs. 5 and 6, the benzene removal is significantly enhanced when limestone is coated with unmodified LAC and when sand is coated with LAC-Fe. Additionally, Fig. 6 showed a higher removal rate of Toluene when using sand coated with LAC-Fe compared to limestone coated with LAC-Fe, despite that both coated materials reached a similar removal efficiency of 40% after 60 min. Moreover, results indicated that, in general, a better removal efficiency of Toluene is achieved when using unmodified LAC-coated materials compared to when using materials coated with Fe-modified LAC.

### The effect of pH on adsorption by LAC-Fe coated on mix bed of limestone and sand

**Figure 7:**
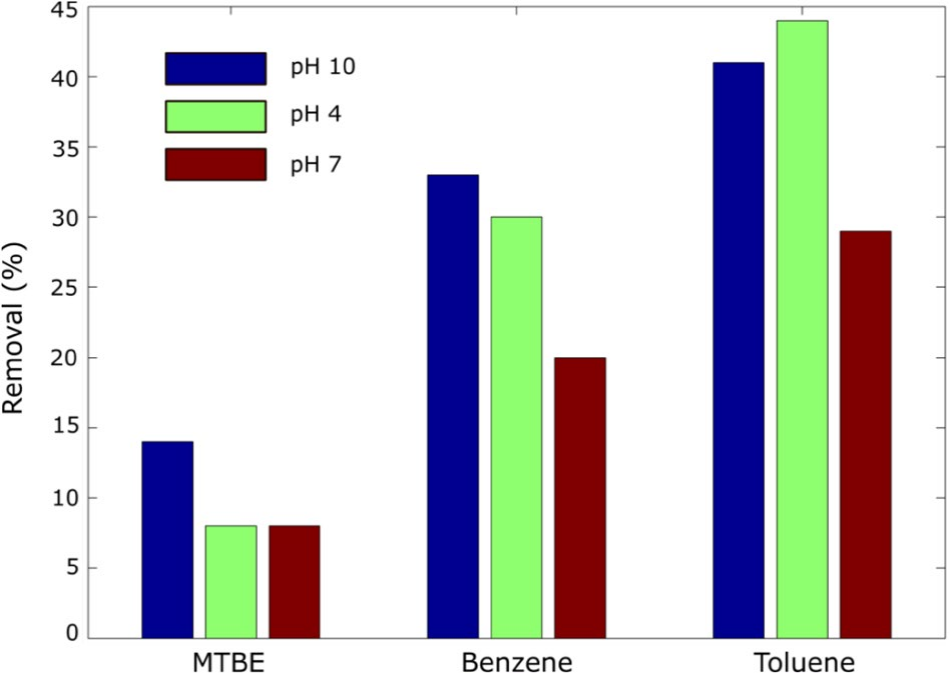
Effect of pH on the removal of MTBE, benzene and toluene using LAC-Fe coated mixed bed.

In an attempt to assess the effect of pH on the removal efficiency of the pollutants, several treatment runs at pH 4, and 10 were carried out using the mixture of limestone and sand of ratio 1:1 coated with Fe-modified LAC (i.e., LAC-Fe). The results are shown in Fig. 7. The results indicate thatchanging the pH from acid to alkaline conditions did not have a major effect on the performance of the LAC-Fe materials in the adsorption of any of the 3 compounds (i.e., MTBE, Benzene, or Toluene), similar with previous studies^33,60–62^. The small differences in removal efficiencies of the 3 pollutants maybe considered within the experimental error.In general, the mixed material coated with LAC-Fe achieved higher removal of Toluene (40%) followed by Benzene (30%), and followed by MTBE with the least removal efficiency of less than 20%. MTBE is less sorptive than Toluene and Benzene, leading to being less competitive to the sorption sites, and requiring longer treatment time. It should be mentioned that, unlike Pump & Treat systems, employing AC in real field applications requires longer residence time (weeks-months). The additional removal of pollutants may have occurred due to the adsorption by LAC-Fe.

### The effect of salinity on adsorption by LAC-Fe coated on mix bed of limestone and sand

**Figure 8:**
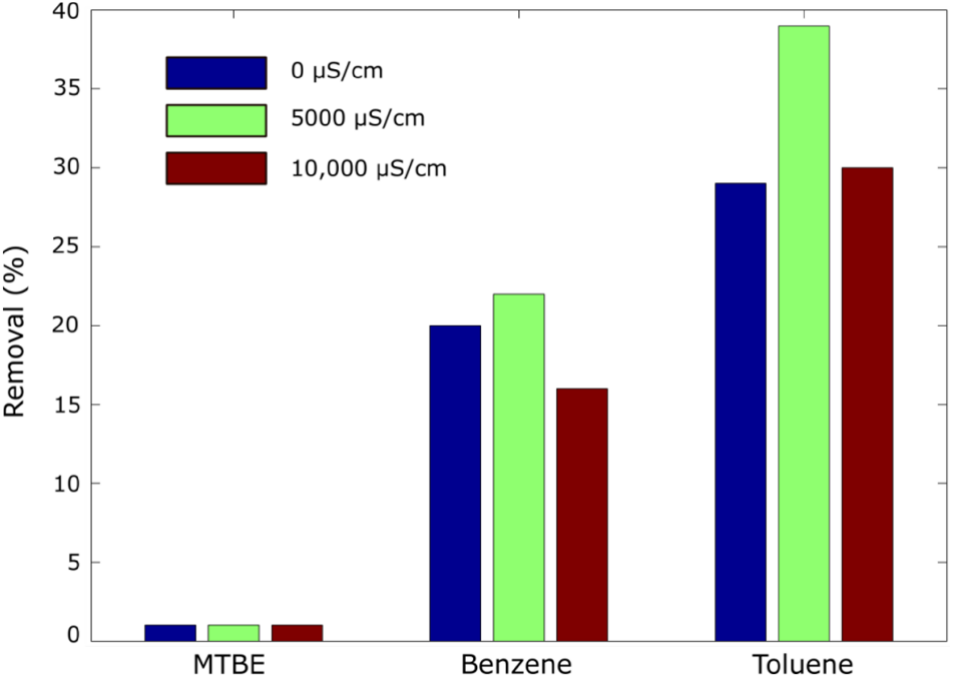
Effect of conductivity on removing MTBE, benzene, and toluene using LAC-Fe coated mixed bed.

Figure 8 presents the results of the treatment runs using spiked water of different salinities. Similar to the treatment runs done in “The effect of pH on adsorption By LAC-Fe coated on mix bed of limestone and sand” section, the 2 ppm spiked samples were treated with a mix of limestone and sand at a ratio of 1:1 coated with LAC-Fe. The results showed a slight decrease in the MTBE, Benzene, and Toluene removal efficiency when the water conductivity was increased from 5000 to 10,000 μS/cm. Nourmoradi et al.^33^ also reported insignificant effect of salinity in BTEX adsortion by modifed montmorillonite. This is expected since the increase in salt loading in the brackish water will occupy some of the voids of the particles of the mix and reduces the chances of target compounds to be adsorped on the surface.

Langmuir and Freundlich isotherm models for selected samples on Benzne and MTBE calculated to test the efficiency of the adsorbent materials and presented on Table 2. Even though, only five points were used for each sample, the experimental data were fitted in the Langmuir (monolayer) and Freundlich (heterogeneous multi-layer) process models. The data were fitted into the Temkin and Dubinin-Radushkevich, however the R^2^ obtained were both < 0.8.

**Table 2:** Parameters of the Langmuir and Freundlich isotherm models on benzenze and MTBE.

	Adsorbent	Langmuir			Freundlich		
		Qm (mg/g)	K_L_ (L g^−1^)	R^2^	n	logK_F_ (mg^1−n^ g^−1^ L^n^)	R^2^
Benzene	GAC	0.881	0.023	0.701	17.85	0.005	0.908
	GAC LAC	2.79	0.104	0.479	2.87	1.49	0.829
	GAC LAC-Fe	1.841	0.044	0.446	2.28	1.59	0.826
Benzene	MT	0.0306	8.06·10^–7^	0.942	0.32	9.57	0.984
	MT LAC	0.355	8.85·10^–5^	0.919	0.322	10.36	0.979
	MT LAC-Fe	0.135	9.53·10^–6^	0.927	0.17	19.54	0.981
MTBE	GAC	0.65	1.029	0.548	1.06	2.66	0.856
	GAC LAC	1.12	0.0047	0.591	1.18	2.74	0.871
	GAC LAC-Fe	0.838	0.0027	0.595	1.06	2.87	0.879
MTBE	MT	N/A	N/A	N/A	N/A	N/A	N/A
	MT LAC	N/A	N/A	N/A	N/A	N/A	N/A
	MT LAC-Fe	0.017	1.82·10^–7^	0.992	0.065	49.28	0.998

## Conclusions

This study demonstrated the utilization of raw and Fe-modified biochar-based liquid activated carbon in removing selected organic water pollutants (MTBE, benzene, and toluene) coated on different solid materials. Overall, the removal efficiencies of these compounds using LAC were lower than that achieved by GAC with benzene and toluene were better removed by LAC and LAC-Fe (∼ 40%) than MTBE (∼20%). Moreover, results revealed that the type of solid materials had a noticeable effect on the removal efficiency of benzene and toluene due to the low affiinity of MTBE to adsorption. Water salinity and pH effect on the removal efficiency was marginal and probably within the experimental error. In conclusion, the adsorption by liquid activated carbon need to be further investigated in terms of enhancing the coating of LAC on the soild substrate, increasing the adsorption time, varying the concentration for adsorbent and de-sorbent, reusability, and long term performance of the prepared LAC. Furthermore, the porosity of adsorbent should be identified in order to determined the exact effect of the porosity size. On sites that have very high contaminat concentrations or where high groundwater flow velocities may “wash out” LAC, carbon size and concentration should be optimized. These parameters are currently investigated aiming to enhance the adsorption of benzene, toluene and MTBE using column adsorption study. It is expected that results of these studies will lead to improve the utilization of liquid activated carbon for the in-situ remediation of contaminated groundwaters and address a number of challenges in the groundwater clean-up sector.

## Data Availability

The datasets used and/or analyzed during the current study are available from the corresponding author on reasonable request.
